# Decoding the CSF Proteomic Signature of Idiopathic Normal Pressure Hydrocephalus: A Systematic Review

**DOI:** 10.3390/molecules31132319

**Published:** 2026-07-02

**Authors:** Aleksandra Kwiecień, Małgorzata Dudzic, Andrzej Lemański, Justin M. Kalka, Artur Drużdż, Katarzyna Hojan, Giorgio Palandri, Bartosz Sokół

**Affiliations:** 1 Department of Neurology, Municipal Hospital in Poznań, 3 Szwajcarska Street, 61-285 Poznań, Poland; olakwiecien0@o2.pl (A.K.); adruzdz@op.pl (A.D.); 2 Department of Neurosurgery, Municipal Hospital in Poznań, 3 Szwajcarska Street, 61-285 Poznań, Poland; alemanski@szpital-strusia.poznan.pl (A.L.); bsokol@szpital-strusia.poznan.pl (B.S.); 3 Faculty of Medicine, Poznań University of Medical Sciences, 10 Fredry Street, 61-701 Poznań, Poland; justin4kalka@gmail.com; 4 Department of Occupational Therapy, Poznań University of Medical Sciences, 6 Święcickiego Steet, 60-781 Poznań, Poland; 5 Department of Rehabilitation, Greater Poland Cancer Center, 15 Garbary Street, 61-866 Poznań, Poland; 6 Institute of Neurological Sciences of Bologna, Scientific Institute for Research, Hospitalization and Care, Bellaria Hospital, 3 Via Altura, 40139 Bologna, Italy; giorgio.palandri@unibo.it

**Keywords:** neurodegenerative diseases, idiopathic normal pressure hydrocephalus, proteomic, CSF proteomics, inflammation, shunting response

## Abstract

Idiopathic normal pressure hydrocephalus (iNPH) is a potentially reversible neurological disorder characterized by gait disturbance, cognitive impairment, and urinary incontinence; however, its diagnosis and prediction of shunt responsiveness remain challenging. This systematic review aimed to synthesize current evidence on cerebrospinal fluid (CSF) proteomic biomarkers in iNPH and to identify molecular patterns with diagnostic and prognostic relevance. A PRISMA-guided search of PubMed, Web of Science, and Google Scholar identified 14 eligible studies comprising 1171 iNPH patients. Proteomic analyses revealed substantial heterogeneity in study design and detected proteins; however, consistent patterns emerged. iNPH is associated with upregulation of inflammatory and extracellular matrix-related proteins and relative downregulation of synaptic and neuronal markers. Neurodegenerative proteins, including amyloid-β, tau, and neurofilament light chain, demonstrated value in differentiating iNPH from comorbid neurodegenerative diseases and in predicting response to ventriculoperitoneal shunting (VPS). These findings support a multifactorial model of iNPH involving impaired glymphatic clearance, neuroinflammation, blood–brain barrier dysfunction, and mechanical axonal stress. Multidimensional biomarker profiles, rather than single proteins, appear to provide the greatest clinical utility, highlighting the need for standardized proteomic panels and integrative predictive models. However, given the substantial heterogeneity of the included studies and the predominantly exploratory nature of current proteomic evidence, the identified proteins should be interpreted as candidate biomarkers rather than clinically validated diagnostic or prognostic tools. Multidimensional biomarker profiles appear biologically plausible and may offer greater explanatory value than single proteins, but their clinical utility requires validation in standardized prospective cohorts. The authors therefore propose a conceptual iNPH proteomic “Vulnerability Model” integrating CSF biomarkers to reflect the balance between reversible and irreversible pathology; this is currently a hypothetical model that requires rigorous statistical and clinical validation through large-scale prospective cohort studies before it can fulfill its potential for improving patient stratification and prediction of postoperative outcomes.

## 1. Introduction

Idiopathic normal pressure hydrocephalus (iNPH) is a neurological condition characterized by the following triad of symptoms: gait disturbance, cognitive dysfunction and urinary incontinence associated with normal cerebrospinal fluid (CSF) pressure on lumbar puncture [1]. The pathophysiology of iNPH remains complex and multifaceted, involving several interrelated mechanisms [2]. The diagnosis of iNPH is challenging due to its symptomatic overlap with other neurodegenerative diseases such as Alzheimer’s disease (AD), Parkinson’s disease (PD) and atypical parkinsonisms [3] or vascular degeneration. Since iNPH is one of the few potentially reversible causes of dementia with ventriculoperitoneal shunting (VPS), accurate differentiation is of paramount importance.

The main challenges in iNPH management include the accuracy of the diagnosis and differentiation with its mimics [4], preoperative care and decision making with the lack of precise surgical responsiveness biomarkers [5]. The current diagnostic protocols for iNPH rely primarily on clinical assessment [6], neuroimaging—particularly magnetic resonance imaging (MRI) and CSF drainage tests [1,7]. However, these methods are often limited by their invasiveness, variability, and lack of disease specificity, and thus do not accurately predict shunt response. In this context, the identification of reliable biomarkers of iNPH has attracted increasing interest due to their potential to facilitate earlier diagnosis, improve differential diagnosis, and provide means to monitor disease progression and response to therapeutic interventions.

Novel biomarkers for iNPH are currently under active investigation. Recent proteomic studies have identified several promising candidates in the CSF that correlate with clinical outcomes, particularly regarding shunt response [3]. Proteomics, the large-scale study of proteins, offers a robust methodology for identifying biomarkers in iNPH. This approach allows researchers to analyze complex protein expressions in CSF, thereby providing insights into the pathophysiology of iNPH and potential diagnostic tools.

Emerging evidence suggests that iNPH may be more appropriately conceptualized as a multifactorial condition arising from the interaction between impaired clearance mechanisms, vascular inflammatory dysfunction, and mechanical axonal stress, in which the clinical response to VPS may depend on the balance between potentially reversible axonal dysfunction and irreversible neurodegenerative processes.

This review aims to synthesize and critically evaluate the current state of research on biomarkers relevant to iNPH, with a particular focus on CSF proteins identified through proteomic approaches. Identifying novel biomarkers and clarifying their clinical utility is essential for improving diagnostic accuracy and informing therapeutic decision making in iNPH. Beyond summarizing individual proteins, the review also seeks to define common pathophysiological mechanisms and propose an integrative model of iNPH with potential clinical application, particularly in predicting shunt responsiveness (SR).

## 2. Results

The review of three databases was performed: PubMed, Web of Science, and Google Scholar. From a total of 61 articles reviewed, 11 duplicates were eliminated. Of the 50 remaining studies, only 14 were included. The remaining 29 papers did not meet the inclusion criteria, and another 7 articles were excluded due to lack of relevance to the research questions’ aim and objectives. The forementioned studies included mostly research without VPS. Table 1 illustrates the group characteristics and the main findings of the studies included in the review. The pooled sample size of these 14 studies was n = 1171 patients with iNPH and n = 764 patients with iNPH that underwent VPS. A substantial heterogeneity in sample sizes was observed across the included studies, with cohort sizes ranging from as few as 24 participants to as many as 354, which may affect the generalizability of the reported biomarker associations. The largest numbers of biomarkers were analyzed in studies using advanced proteomic panels and mass spectrometry. The study by Kamalian et al. [1] used the Olink Explore 3072 panel, which allowed for the identification of 2888 unique proteins in the CSF. In the study by Weiner et al. [2], 2795 proteins were identified, of which 1860 could be reliably quantified in most patients. Ying et al. [3] identified a total of 2762 proteins, of which 1684 were detected in all samples tested. Research by Rostgaard et al. [4,5] identified 1251 and 1231 unique proteins in ventricular and lumbar CSF, respectively. The study by De Geus et al. [6] generated a dataset comprising 576 unique protein groups. Other studies focused on a smaller, strictly defined number of markers. The study by Braun et al. [7] analyzed a panel of 92 proteins associated with inflammation, 56 of which were detectable in more than 75% of participants. The works of Lukkarinen et al. [8,9] focused on a panel of core biomarkers for AD and iNPH, including: amyloid-β 42 (Aβ42), amyloid-β 40 (Aβ40), amyloid-β 38 (Aβ38), total microtubule-associated protein tau (T-tau), phosphorylated microtubule-associated protein tau 181 (P-tau181), neurofilament light polypeptide (NfL), neurogranin (NRGN). Two studies focused on a detailed analysis of one main marker: neuronal pentraxin 2 (NPTX2) [10] and Aquaporin 4 (AQP4) [11]. As a result of the above analyses, some of the authors identified smaller sets of proteins with the greatest diagnostic significance. Kamalian et al. [1] proposed a predictive panel of thirteen proteins that distinguishes iNPH from MCI and the control group. De Geus et al. [6] created a panel of twelve proteins with high diagnostic potential for iNPH identified by machine learning algorithms. Weiner et al. selected nine candidates for predictive biomarkers of response to shunting.

The quality assessment of the included studies is presented in Table 2. The weak global ratings were primarily driven by two factors. First, there was a lack of reported blinding. Second, the study design was non-randomized. These are inherent limitations of exploratory biomarker studies, like the included papers.

## 3. Discussion

The application of proteomics in the investigation of iNPH has emerged as a promising avenue for enhancing diagnostic accuracy and elucidating the underlying pathophysiological mechanisms of this complex condition. Recent studies have identified discrete proteomic profiles in the CSF of iNPH patients, revealing substantial alterations that may differentiate it from other neurodegenerative diseases. Furthermore, the authors of some discussed studies analyzed the dynamics of protein increase/decrease after VPS, identifying several promising candidates as predictors of therapeutic success of the procedure.

It should be emphasized that the current proteomic evidence in iNPH remains primarily exploratory. The included studies differ substantially in cohort size, CSF sampling site, proteomic platform, statistical approach, comparator groups, and definitions of postoperative improvement. Consequently, the proteins discussed in this review should not be interpreted as established clinical biomarkers, but rather as candidate markers with varying degrees of biological plausibility and preliminary prognostic relevance. Their value lies mainly in identifying convergent pathophysiological pathways, including neuroinflammation, impaired CSF–interstitial fluid exchange, blood–brain barrier dysfunction, extracellular matrix remodeling, synaptic dysregulation, and axonal stress. Whether these candidate biomarkers provide incremental predictive value beyond established clinical, radiological, and CSF drainage-based assessments remains to be determined in adequately powered prospective studies.

To systematically structure current knowledge on the pathophysiology of iNPH and to explain the role of specific proteins within the cascade of events underlying its development, the investigated proteins were classified into five principal categories: inflammatory proteins, neurodegeneration-associated proteins, synaptic and neuronal markers, cell adhesion molecules, and others. However, it should be noted that this is an artificial division, as many proteins perform several different functions, and the function of some proteins is still not fully understood. To further conceptualize the potential pathophysiology of iNPH, a biologically grounded integrative model has been proposed, suggesting links between reversible and irreversible pathophysiological mechanisms and SR.

### 3.1. Inflammatory Pathways

The role of inflammatory proteins in the cascade of events leading to the development of iNPH is not completely understood. However, recent research suggests that disturbances in CSF are accompanied by chronic neuroinflammation. This is a complex phenomenon in which blood–brain barrier (BBB) leakage, complement activation, and a specific cytokine response play a key role. As demonstrated in the analysis of CSF test results, the upregulation of acute phase proteins, complement, and immunoglobulins is characteristic of iNPH [14].

A comparative analysis of CSF of patients diagnosed with iNPH revealed elevated levels of specific pro-inflammatory cytokines, including MCP-1 (CCL2) and CCL4, when compared to the CSF of healthy subjects. Furthermore, the levels of these proteins were found to be significantly higher in the iNPH group compared to patients with mild cognitive impairment (MCI), AD, and frontotemporal dementia [7]. In addition, PD-L1 levels were found to be slightly lower in patients with iNPH compared to patients with MCI and AD. In the case of frontotemporal dementia, PD-L1 levels were reduced, though not to the same extent as in iNPH. The aforementioned facts may be of clinical significance, particularly given that iNPH has the capacity to manifest symptoms that are similar to those of the above-mentioned disease entities. MCP-1 plays an important role in the activation of monocytes/macrophages and the recruitment of macrophages, microglia cells, and Th lymphocytes, leading to the accumulation of these cells in the choroid plexus, which is responsible for the production of CSF. MCP-1 is produced by various cells, including fibroblasts, smooth muscles, and in the nervous system, primarily astrocytes, monocytes, and microglia cells. Its association with oxidative stress is well-documented, and its levels are known to increase in a variety of diseases, including neurodegenerative diseases [7]. Elevated MCP-1 levels have been reported in traumatic brain injury and the early stages of AD, where an initial increase is followed by a decrease [7]. CCL4 is predominantly produced by macrophages and plays a pivotal role in the infiltration of inflammatory cells into the CNS. It has been observed to affect endothelial cells and is associated with BBB damage. Its elevated levels have also been observed in other CNS diseases, including multiple sclerosis, PD, and AD [15,16,17]. The PD-L1 protein binds directly to the PD-1 receptor, which is located on the surface of various immune system cells, including T lymphocytes, B lymphocytes, macrophages, and dendritic cells. This interaction results in the transmission of an inhibitory signal to the interior of the immune cell, thereby reducing the effector activity of the immune system. Reduced PD-L1 levels in the CSF of patients with iNPH indicate an intensified neuroinflammatory process. Interestingly, reduced levels of this protein were also observed in patients with frontotemporal dementia, while in patients with MCI and AD, the level of this protein was elevated [18].

Proteomic studies have also demonstrated that iNPH is associated with robust upregulation of the complement cascade and acute phase proteins [14]. In the CSF of iNPH patients, a wide range of complement system proteins involved in the development of the disease have been identified, including C1QA, C1QB, C1QC, C1R, C1S, C2, C3, C4A, C4B, C5, C6, C7, C8B, C8G, and C9 [6]. The C1 protein is likely responsible for initiating the cascade, leading to an increase in C6 and C8 levels, which prevents them from forming the C5b67 complex. The C5b67 complex is a stage of the complement cascade that, under physiological conditions, leads to the formation of the membrane attack complex (MAC). The MAC is responsible for cell lysis. This finding indicates that, in iNPH, C6 and C8 may be utilized in alternative inflammatory or signaling processes, rather than participating in the conventional lytic complex. An intriguing hypothesis was advanced by Bode et al., proposing a potential association between C1q and ceramide transferase in the context of ceramide transport and apoptosis processes. According to the findings of this research, C1q appears to play a role in preventing autoimmunity and maintaining self-tolerance by promoting the efficient removal of apoptotic cells [19].

The literature indicates that the protein profile of iNPH is unique [1,6]. While iNPH is dominated by strong upregulation of immune system processes, in AD these proteins often show lower levels or are involved in other pathways [6]. In iNPH, an inflammatory profile associated with vascular and endothelial damage predominates, while in AD, high levels of C8 and C9 components are associated with chronic neurodegeneration. There is a suggestion that chronic inflammation may increase the risk of initiating neurodegeneration. Therefore, the levels of C8, C9, and fetuin-B (FETUB), along with the levels of neurodegeneration proteins, should be measured not only for differential diagnosis but also monitored to assess the likelihood of parallel development of AD [14]. The increased presence of complement proteins and immunoglobulins in the CSF of iNPH patients is directly related to BBB leakage and inflammatory cell infiltration, which is less common in such severe forms in the early stages of AD [14].

Increased levels of acute phase proteins such as FETUB, haptoglobin (HP), alpha-1-antichymotrypsin (SERPINA3) and fibrinogen also indicate an intense neuroinflammatory process [14]. FETUB is a protein that performs a number of functions in the body: it is a metabolic protein, plays a role in the pathogenesis of type II diabetes and NAFLD (non-alcoholic fatty liver disease), and its relationship with both inflammatory and neurodegenerative processes of CNS is significant. An increase in FETUB has been observed in both iNPH and AD, which is why it is important to correlate the level of this protein with the levels of other inflammatory proteins (such as C6, C8, and C9) and neurodegenerative proteins (neurocan, brevican). SERPINA3 is the main driver of the acute phase response signaling pathway. Upregulation of this protein is characteristic of the molecular profile of iNPH, while downregulation is typical of the molecular profile of AD, making it a useful tool in the differential diagnosis of these disease entities [14]. Another protein, TIMP-1 (tissue inhibitor of metalloproteinases-1), plays an important role in regulating the balance between synthesis and degradation of the extracellular matrix (ECM); it can both bind to metalloproteinases (MMPs), blocking their action and preventing the destruction of the ECM, and promote cell proliferation in various types of tissue. MMPs and TIMP-1 play a key role in the immune response, and their dysregulation occurs in many inflammatory, autoimmune, and neoplastic diseases [13]. In the context of iNPH, it is important to note that, similar to SERPINA3 protein levels, TIMP-1 levels are elevated in patients with iNPH and decreased in patients with AD.

Analysis of the CSF inflammatory profile may also help predict the response to VPS. Patients who responded well to surgical treatment showed higher activity of IKK/NF-κB inflammatory pathways and proteins such as MIF (macrophage inhibitory factor) prior to surgery. In turn, high levels of inflammatory proteins associated with vascular damage may indicate a more advanced, irreversible pathological condition in which the chances of improvement after VPS are lower [3].

### 3.2. Neurodegenerative Proteins

Neurodegenerative proteins in iNPH serve as biomarkers for brain tissue damage assessment, differentiation from other neurodegenerative diseases (e.g., AD), and prediction of the VPS response. As mentioned earlier, iNPH is a potentially reversible cause of dementia, but many patients have comorbidities that are irreversible, which significantly affects the clinical picture, prognosis, and chances of a favorable response to shunting. The typical neurodegeneration protein profile in iNPH is as follows: low levels of APP and tau, high levels of NfL [9]. In iNPH, there are disturbances in the function of the glymphatic system, which is responsible for brain detoxification by removing unnecessary metabolic products, including proteins. One of the proteins that accumulates in the brain tissue of patients with iNPH is Aβ, which occurs in many forms that differ in chemical structure, toxicity, and aggregation ability. The Aβ-40 and Aβ-42 forms differ from each other by only two amino acids, but despite such a small difference in structure, their properties are significantly different. Aβ-42 is much more prone to form aggregates and accumulate in brain tissue as amyloid plaques, and is also considered to be a more neurotoxic form. The ratio of the above-mentioned amyloid forms to each other is important in the diagnosis of neurodegenerative diseases. In AD, there is a characteristic decrease in the level of Aβ-42 in the CSF associated with the accumulation of this protein in brain tissue, and consequently a decrease in the Aβ-42/Aβ-40 ratio. If this ratio is low in iNPH patients, it is likely that Alzheimer’s pathology is also present. A typical profile in iNPH is characterized by a high Aβ-42/Aβ-40 ratio and a lower level of APP (the precursor protein of all forms of amyloid) than in AD, which may be associated with reduced brain metabolism in the periventricular zone and/or result from dilution of this protein concentration due to an enlarged ventricular system [13]. Aβ-42 is also a good predictor of response to shunting; preoperative concentration of this protein in CSF correlates with improvement in cognitive function assessed in Mini-Mental State Examination (MMSE) and gait efficiency [9].

T-tau (total tau protein) is a marker of general neuronal damage and breakdown with often lower levels in iNPH than in AD. Preoperative T-tau concentration is one of the best predictors of VPS. Patients with low concentrations of this protein showed significant improvement in walking speed after surgery and were also more likely to score high on cognitive tests (≥26 points on the MMSE scale) before and after surgery [9]. The postoperative increase in the level of this protein (by as much as 140–810%) in CSF may be related to the course of the disease itself, damage caused by neurosurgical intervention, or more effective protein removal as a result of improved CSF drainage after valve implantation [9]. P-tau (phosphorylated tau protein) is a specific marker of Alzheimer’s pathology; high concentrations of this protein suggest coexisting AD, which is usually associated with a poorer response to shunting [9]. The level of this protein correlates with the level of another protein, NPTX2.

NfL protein is a component of the cytoskeleton of neurons, and its presence in CSF suggests neuronal damage. In the iNPH, the increase in the level of NfL results from stretching, compression, and destruction of the axons of large, myelinated neurons in front of the enlarged ventricular system. Importantly, in contrast to most neurodegenerative disorders, elevated NfL in iNPH may reflect a potentially reversible form of axonal injury rather than irreversible degeneration. High baseline NfL levels may correlate with a better response to shunting; a transient increase in this protein is observed after surgery, probably due to damage to neurons during valve implantation, after which the level normalizes within 6–9 months [8].

### 3.3. Cell Adhesion Proteins

Given the presence of BBB impairment in iNPH, cell adhesion proteins are significant in pathophysiology, with their CSF profile characterized predominantly by a significant downregulation. The BBB is a highly selective, semipermeable border formed by endothelial cells, which meticulously regulates the passage of solutes and chemicals between the bloodstream and the CNS. This function is essential for protecting the brain from potentially harmful blood-borne substances [20]. The integrity of the BBB relies on a complex of endothelial cells, pericytes, and astrocytic endfeet. This cellular complex controls molecular exchange, a process governed by adherent junctions. Therefore, cell-adhesion markers, e.g., cadherin 5 (CDH5), are fundamental to maintaining the barrier’s function. De Gaus et al. [6] suggest that the downregulation of CDH5 in iNPH is indicative of a loss of BBB integrity in this condition. Furthermore, other proteins such as TGFBI and ITGB1 have also been implicated in modulating BBB permeability [2].

The disruption of BBB integrity results in the leakage of plasma proteins, such as fibrinogen and other blood-derived neurotoxic proteins (e.g., fibrin, thrombin, hemoglobin, and free iron), as well as various inflammatory mediators into the brain parenchyma. Once in the CNS, these proteins accumulate, contributing to the pathology of the disease through progressive neurodegeneration and neuron loss mediated by direct neuronal toxicity, oxidative stress, and the detachment of neurons from their supporting extracellular matrix [21].

The importance of cell junctions in maintaining CNS homeostasis is further underscored by research on hydrocephalus development. Studies have highlighted the critical role of various tight and adherens junction proteins, most notably N-cadherin (CDH2), in preserving the integrity of the neuroepithelium and supporting glial progenitor cells. Disruptions to these proteins have been linked to the development of hydrocephalus [1,22].

Among the protein markers showing high diagnostic potential for differentiating iNPH from other conditions, latent-transforming growth factor beta-binding protein 2 (LTBP2) is believed to play an integral structural role in the organization and assembly of elastic fibers [6]. It was found to be significantly elevated in the CSF of individuals with iNPH when compared to cognitively unimpaired controls and patients with AD.

The apparently conflicting findings on vimentin may reflect differences in disease stage, sampling site, and the extent of ependymal injury rather than a true biological contradiction. Upregulation of vimentin in the iNPH CSF, as reported in some studies [1], may indicate active ependymal stress or denudation, particularly in patients with ongoing ventricular wall remodeling, whereas reduced levels, as described by Rostgaard [4], may correspond to cohorts with less pronounced structural disruption or a phase in which acute ependymal activation is no longer prominent. Therefore, vimentin should be interpreted as a context-dependent marker of ependymal response rather than a universal feature of iNPH. When considered alongside endothelial markers such as CD9, CD81 and Vascular cell adhesion protein 1 (VCAM1), these findings support a broader pathophysiological framework in which ependymal injury, vascular inflammation, and blood–brain barrier (BBB) dysfunction represent interrelated but variably expressed components of iNPH [3,12].

To understand the mechanism of neuroepithelium and synapse damage in iNPH, it is important to understand that cadherins are calcium-dependent proteins that are binding cells together to preserve tissue structure (e.g., E-cadherin in epithelial barriers). When ependymal cells lining the brain ventricles undergo apoptosis, denudation of the ventricular wall occurs. This disrupts cadherin bonds, compromising the BBB and exposing brain tissue to CSF.

In iNPH, reduced levels of neuron-specific adhesion molecules—such as L1 cell adhesion molecule (L1CAM), contactin-associated protein-like 2 (CNTNAP2), cadherin EGF LAG seven-pass G-type receptor 2 (CELSR2), and various cadherins (e.g., PCDH7, PCDH9, CDH2)—have been reported. Kamalian et al. emphasize a significant downregulation in cell–cell adhesion processes in iNPH [1]. The dysregulation of neuron-specific adhesion molecules crucial for axonogenesis and synaptic maturation (e.g., CNTNAP2, CELSR2, PCDH7, PCDH9, PCDH17, CDH2, CDH6, and CDH15) suggests impaired neuroepithelial integrity and potentially disrupted neuronal connectivity. Moreover, the downregulation of polycystin 1 (PKD1) and CELSR2—both linked to ependymal planar polarity and CSF circulation—aligns with established hypotheses of hydrocephalus pathogenesis.

A particularly important protein in this context is L1CAM. Downregulation of L1CAM, a molecule associated with congenital hydrocephalus, points to a potentially shared molecular basis between congenital hydrocephalus and iNPH. Kamalian et al. [1] identified downregulation of specific genes, including L1CAM, which has been well characterized for its role in X-linked congenital hydrocephalus and aqueductal stenosis. This finding is especially noteworthy given that prior studies have linked mutations in the CWH43 gene in iNPH patients to increased L1CAM proteolysis. In conclusion, their comprehensive CSF proteomic analysis comparing iNPH with MCI and healthy controls revealed distinct patterns in iNPH, including downregulation of synaptic markers and proteins essential for cell–cell adhesion (e.g., L1CAM, DSCAM, various cadherins) and ependymal planar polarity, alongside upregulation of vimentin—suggesting ependymal denudation and dysfunction. Furthermore, the parallels drawn with congenital hydrocephalus (e.g., L1CAM involvement) offer a new perspective on possible shared molecular mechanisms.

Although neuronal pentraxins (NPTX1, NPTX2, NPTXR) are not part of the classic adhesion families (such as cadherins or integrins), these are proteins with adhesive and organizing functions at synapses, and represent a distinct, specialized class of “synaptic organizers.” Torretta et al. reported that levels of the neuronal pentraxin receptor (NPTXR) and L1CAM were higher in iNPH than in AD [14]. In addition, NPTX1—a protein involved in synaptic function—was differentially expressed in iNPH patients compared to cognitively healthy individuals. NPTX2 is an established biomarker of synaptic integrity. According to Ivarsson et al. [12], NPTX2 levels are known to change in AD. Patel et al. [10] demonstrated that although CSF NPTX2 levels correlate with neurodegeneration, they do not correlate with cognitive or functional measures in iNPH. Specifically, NPTX2 was not a reliable predictor of short- or long-term improvement after CSF drainage, nor did it predict outcomes following shunt surgery. The apparent absence of extensive synaptic injury in iNPH supports the view that iNPH is primarily a subcortical process and may explain the reversibility of cognitive impairment due to preserved synaptic structure.

The potential dysfunction of the glymphatic system—a crucial paravascular pathway for clearing toxic waste and maintaining biochemical homeostasis in the brain—has emerged as a significant factor in the pathogenesis of iNPH [1,6,13]. Several hypotheses have been proposed to explain the clinical decline in iNPH, among which impaired glymphatic circulation and a reduced flow in the interstitial fluid (ISF) space are increasingly recognized [7,13]. This system is essential for the clearance of peptides and neurotoxic waste from the cerebral ISF to the CSF, and its disruption could explain the inadequate drainage of neuronal markers, contributing to their decreased presence in the CSF [1]. Such impaired clearance may in turn contribute to cognitive dysfunction [10]. Furthermore, given that the BBB and the glymphatic system are anatomically and functionally interconnected, alterations in related proteins may reflect underlying glymphatic dysfunction [2]. This hypothesis is supported by imaging studies indicating reduced glymphatic activity in iNPH, which aligns with observed synaptic and neuronal marker downregulation [1].

Kamalian et al. further observed downregulation of several synaptic and neuronal markers in iNPH, including neuronal pentraxins and their receptor (NPTX1, NPTX2, NPTXR), neurogranin (NRGN), synaptotagmin-1 (SYT1), and synaptosomal-associated protein 25 (SNAP25), all of which are integral to synaptic function and neuronal communication [1]. Rather than reflecting purely structural synaptic loss, the reduced CSF concentrations of these proteins may indicate a secondary disturbance in neuronal signaling associated with impaired glymphatic clearance and metabolic dysfunction, particularly when considered alongside disrupted ISF–CSF exchange. Given their role in axonogenesis and synapse maturation, their deficiency likely contributes to impaired neuronal connectivity, while additional factors such as mechanical compression and ischemia may further exacerbate deficits in signal transmission underlying the cognitive features of iNPH [10]. Nevertheless, despite deficiencies in signaling proteins, synaptic structure in iNPH appears largely preserved, with no primary cortical neurodegeneration. This may account for the potential reversibility of symptoms following VPS. Decompression of hydrocephalus can restore normal metabolic dynamics and improve neuronal connectivity, clinically reflected in better gait and cognitive function among treatment responders.

Moreover, protocadherin alpha subfamily C2 (PCDHAC2) may serve as a biomarker distinguishing communicating from obstructive hydrocephalus. In a comparative proteomic analysis, CSF from patients with communicating hydrocephalus, obstructive hydrocephalus, and healthy controls showed distinct profiles. Specifically, PCDHAC2 was found in significantly higher abundance in healthy controls than in patients with communicating hydrocephalus, suggesting its potential utility as a diagnostic marker [4].

Fibronectin (FN1) is a protein that binds cell surfaces and various compounds, including collagen, fibrin, heparin, actin, and DNA. It plays a role in both cell adhesion and the structural integrity of the glymphatic system. Recent findings suggest that fibronectin may serve as a potential marker of treatment response in obstructive hydrocephalus, with levels significantly lower in the shunt-responder group compared to non-responders [4].

Adhesion molecules also serve as precise clinical indicators. For example, Desmoglein-2 (DSG2) is a desmosomal adhesion protein that plays a critical role in maintaining tissue integrity through cell–cell junctions. Recent proteomic studies have identified DSG2 as a promising prognostic biomarker in iNPH. Both Weiner et al. [2] and Ying et al. [3] demonstrated that DSG2, along with ITGB1, YWHAG, OLFM2, and TGFBI, was among the top five CSF biomarkers most strongly correlated with improvement in gait speed one year after VPS.

Beyond its prognostic value, DSG2 may also hold diagnostic relevance. It has also been implicated as a genetic risk factor for AD, suggesting potential overlap in molecular pathways between iNPH and neurodegenerative disorders. It is worth mentioning that DSG2 is the only marker that correlates with all three of the triad of symptoms and the severity of the disease.

### 3.4. Synaptic and Neuronal Markers

Neuronal markers are proteins which have been selectively expressed in neurons and synaptic markers and are located in proximity to synapses. From our article selection, we identified synaptic and neuronal markers found to have important changes in patients with iNPH.

A pattern seen in multiple studies, and of significant interest, is the decrease in synaptic and neuronal markers [1,14,23]. Consistent lowered synaptic and neuronal markers in the existing literature may be explained by disruption in synaptic integrity and neuronal signaling leading to clinical manifestation [1]. It should be considered that although synaptic and neuronal markers may have decreased prevalence in iNPH, the total CSF protein remains constant compared to controls [6,24]. Total protein levels in iNPH patients are comparable to controls, and this is theorized to be from upregulation of alternative protein categories [6].

Synaptic processes downregulated in iNPH seemed to be driven by neuronal adhesion molecules such as neural cell-adhesion molecule 1, neurexin 1, neurexin 3 (NRXN3), and axon guidance proteins from the semaphorin family [6]. The term “Tight junction” (Zonula Occludens), which regulates paracellular permeability between adjacent cells, was among the significantly downregulated terms in iNPH. Similar synaptic membrane-related sets of proteins were found to be significantly downregulated in iNPH when compared to MCI [1].

Postoperative VCSF found 16 proteins, associated with axon development, upregulated after VPS, which have been downregulated preoperatively. Additionally, proteins involved in astrogliosis are shown to be higher than control, including GFAP and NfL. LINGO1 is a regulator of Ca^2+^-activated K^+^ channels which has been increased in Parkinson’s but decreased in iNPH, which may play a role in neurodegenerative disease differentiation. Vacuolar protein sorting 10 domain-containing receptor (SORCS1) indicated the highest correlation with urinary symptoms of the iNPH triad [3].

The synaptic proteins beta-secretase 1 (BACE1), APP, seizure 6-like protein (SEZ6L), seizure-related 6 homolog-like 2 (SEZ6L2), and neurosecretory protein VGF were described to have statistically lower concentrations in iNPH. Of these proteins active at the synaptic terminals, several showed inverse proportions, where proteins were downregulated in iNPH and upregulated in AD compared to control, including APP and seizure 6-like protein (SEZ6L). The same trend was found in synapse adhesion molecules, proteins involved in axonogenesis and in signal transduction, and for neurosecretory proteins such as chromogranin-A (CHGA), serotonin-3 (SCG3) and neurosecretory protein VGF [14]. This group of proteins adds to the hypothesis that AD and iNPH are interlinked and share some pathophysiological pathways.

On the contrary, several proteins were greater in iNPH than in AD, as previously discussed above in Cell Adhesion. Also not mentioned is that Thy-1 membrane glycoprotein (THY1), 14-3-3 protein zeta/delta (YWHAZ), reticulon-4 receptor (RTN4R), secretogranin2 (SCG2) were upregulated in iNPH [14]. It is important to understand what makes these proteins different from other documented neuronal/synaptic markers in the disease process and further research should address this disparity.

NPTX2 is a synaptic protein responsible for modulating plasticity at excitatory synapses. A significant positive correlation was found between CSF NPTX2 and CSF pTau-181 concentrations. And weak significant negative correlations were found when comparing CSF NPTX2 values to AB42/AB40 ratio and FAQ scores [10]. The study found no correlation between CSF NPTX2 concentration compared to baseline MoCA test score and age. Neurodegenerative comorbidities are playing a greater role in treatment decisions for patients with iNPH [10]. AD is a common comorbidity for patients with iNPH and the literature correlates higher NPTX2 concentrations with neurodegeneration. Because NPTX2 does not correlate to cognitive impairment in iNPH, NPTX2 plays a potential role in the assessment of comorbid neurodegenerative conditions.

### 3.5. Other Proteins

Several proteins have been associated with iNPH pathophysiology but do not align with the predetermined categories.

Fatty Acid Binding Protein 3 (FABP3) plays a role in the intracellular transport of long-chain fatty acids and their acyl-CoA esters. It is involved in lipid metabolism, with particular functions in neuroprotection and energy metabolism, and may also interact with retinol-binding protein 4 (RBP4), potentially influencing neurometabolic and neuroprotective processes [3]. In a study by Ying et al. [3], FABP3 and annexin A4 (ANXA4)—a calcium/phospholipid-binding protein involved in membrane fusion and exocytosis—were identified as two of four CSF proteins strongly associated with clinical improvement in iNPH. Weiner et al. also ranked FABP3 and ANXA4 among the top ten biomarker candidates for predicting SR one year post-surgery. The authors suggest that high FABP3 concentrations may indicate severe brain injury, thereby reducing the likelihood of significant neurological recovery or benefit from shunting. In addition, FABP3 appears to be associated with AD. Several studies have reported significantly increased CSF levels of FABP3 in AD patients compared to healthy controls. Weiner et al. observed that FABP3 concentrations appeared to increase with Aβ and tau positivity, further supporting its potential involvement in AD-related pathological processes.

Proteins involved in lipid metabolism, such as apolipoprotein H (APOH) and lecithin-cholesterol acyltransferase (LCAT), were found to be statistically lower in iNPH patients. In contrast, apolipoprotein E (APOE) is typically elevated in AD.

A distinct pathophysiological mechanism in iNPH may also involve vascular dysfunction, as suggested by two proteins: RBP4 and matrix remodeling-associated 7 (MXRA7). Both were identified as significant candidate biomarkers for predicting postoperative outcomes. RBP4 was found to be decreased in non-responders to shunt surgery [3].

Coagulation factor V (F5), a protein involved in hemostasis, was significantly upregulated in iNPH compared to cognitively unimpaired individuals and AD patients. As noted by De Geus et al. [6], BBB disruption may lead to the extravasation of fibrinogen and other coagulation factors, including F5. Rostgaard et al. reported that F5 levels were significantly higher in the CSF of iNPH patients who responded to shunting compared to non-responders.

The involvement of signaling proteins in iNPH pathology has also been demonstrated. Ying et al. observed that beta-1,4-glucuronyltransferase 1 (B4GAT1) was significantly elevated postoperatively in the responder group and reduced in non-responders. Notably, B4GAT1 upregulation in responders and downregulation in non-responders was associated with recurrent severe ventriculomegaly.

Finally, secretory proteins such as chromogranin-A (CHGA) and secretogranin-3 (SCG3) were found to be statistically lower in iNPH patients, suggesting possible alterations in neurosecretory function [1].

According to the findings of the present study, iNPH may be conceptualized not only as a disorder of CSF circulation, but rather as a disease defined by complicated interaction between impaired clearance mechanisms, vascular inflammatory dysfunction, and mechanical axonal stress. Within this framework, the clinical response to shunting appears to depend on the balance between reversible axonal dysfunction and irreversible neurodegenerative processes.

#### 3.5.1. Role in Shunt Responsiveness Prediction

Of the 14 articles selected, 9 articles included shunting responsiveness correlated to protein concentrations [11]. AQP4 levels did not differ significantly in preoperative iNPH compared to controls. There was, however, a positive correlation to AQP4 levels for both storage duration (r = 0.30, *p* < 0.001) and age (r = 0.28, *p* < 0.001) in iNPH patients compared to controls. During follow-up of iNPH post-shunt response, the authors observed increased AQP4 levels 6 months after surgery. Preoperative AQP4 levels were lower in shunt responders. Although AQP4 concentrations may predict the shunt response, the increase in AQP4 levels post-operatively was weakly positively correlated with a positive shunt response in this study. AQP4 levels in CSF are also elevated in AD and frontotemporal dementia, although they remain within the normal range; this further supports that patients with AD and iNPH may be predictive of poor shunting response. iNPH patients with a lack of diagnosed, or underlying, neurodegenerative comorbid conditions have improved shunting response [11].

Absence of the APOE e4 allele predicted gait improvement post-surgery. Preoperative MMSE results correlated negatively with T-tau, P-tau181, and NfL. Patients with low P-tau181, T-tau, NfL, AB38 and AB40 were more likely to have MMSE scores above 26 compared to higher-concentration groups. P-tau181 was the best predictor of preoperative MMSE scores [9]. P-tau181 and T-tau may be used to predict post-operative cognition [9,11,25,26]. Postoperative MMSE values correlated negatively with P-tau181, T-tau and NfL, and positively with AB42. P-tau181 also positively correlated with AB42 concentrations preoperatively, when the APOE genotype negatively correlated with specifically AB42. NfL showed a negative correlation with preoperative gait velocity, whereas postoperative gait velocity was negatively correlated with T-tau, P-tau181 and NfL. Patients with low T-tau were 3.1 times more likely to improve their gait post-surgery.

The positive correlation of T-tau and P-tau levels in AD has been well documented [11,25,27]; however, the correlation between iNPH and AD comorbidity on shunt response should be further researched. AD may be improperly treated as iNPH with unsuccessful surgical shunting on cognition. Given the correlation between P-tau181 and AD it should be considered whether the underlying iNPH or AD is the primary cause of decrease in cognition [9]. This is further supported by a lack of MMSE change in high preoperative P-Tau concentration compared to low preoperative P-Tau concentration, which correlated with post-shunt improvement in MMSE scores [9].

Brevican and neurocan are proteoglycans expressed exclusively in the brain and spinal cord and play a major role in brain plasticity by regulating cell migration and axonal growth. The concentrations of these two ECM proteins increased in CSF following shunt surgery. Additionally, ventricular CSF MMPs and TIMP-1 showed a postoperative increase. However, none of the ECM proteins in preoperative lumbar or ventricular CSF showed any differences between patients that improved after surgery and those who remained unchanged [13]. The absent correlation between concentration changes pre- and post-operatively brings us to the conclusion that these ECM proteins should be excluded from predicting shunting responses. These protein findings also suggest ECM proteins do not play a primary role in the pathophysiology of iNPH.

NPTX2 has been hypothesized to play a role in iNPH and has been found to have a weakly significant correlation to performance on the Timed Up and Go (TUG), although there was no significant correlation found between CSF NPTX2 and post-shunt surgery TUG performance [10].

Another article found 11 significantly increased proteins and 26 significantly decreased proteins in responders compared to non-responders. CSF QPCT, RBP4, and A8MTW9 were significantly correlated with iNPHG improvement [3,14]. Preoperative CSF presented significantly increased QPCT and decreased RBP4 in non-responders. RBP4 has been implicated in vasculitis and chronic inflammation. VCAM 1 levels were higher compared to HC preoperatively, indicating another predictive protein. Beta-1,4-glucuronyltransferase 1 (B4GAT1), Xyloside xylosyltransferase 1 (XXYLT1), cartilage acidic protein 1 (CRTAC1) concentrations increased in responsive groups and decreased in non-responsive groups [3]. One study found IKK/NF-kappaB signaling, a pathway in inflammatory regulation, was higher in responders than non-responders. One study found clinical improvement correlation with FABP3, ANXA4, MIF, and B3GAT2. MIF is a factor closely associated with neuroinflammation, including tumor suppression. If treated early, before immune function becomes diminished, a functioning immune system may recover post-surgery and begin reversal of prior disease progression. If the immune system cannot maintain its function, the implication of surgery becomes less viable or insignificant in iNPH patients [3].

Adhesion molecules also serve as precise clinical indicators. For example, DSG2 is a desmosomal adhesion protein that plays a critical role in maintaining tissue integrity through cell–cell junctions. Recent proteomic studies have identified DSG2 as a promising prognostic biomarker in iNPH. Several authors [2,3] demonstrated that DSG2, along with ITGB1, YWHAG, OLFM2, and TGFBI, was among the top five CSF biomarkers most strongly correlated with improvement in gait speed one year after shunt surgery. ITGB1 and B3GAT2 have been shown to be strong predictors of response to surgical treatment [2].

The protein markers Aβ, P-tau, T-tau, NfL, and leucine-rich alpha-2-glycoprotein show the strongest evidence in SR for iNPH patients. Also, a number of inflammatory markers, such as interleukins, seem to be associated with a lack of benefit from shunting [4].

Several studies concluded that existing biomarkers for iNPH cannot differentiate comorbid conditions and novel biomarkers are required to determine iNPH with and without AD [4,11,12,28]. iNPH is commonly complicated by comorbid neurodegenerative disorders such as subcortical vascular dementia, parkinsonism, and AD, further supporting that treatment options should consider the coexistence of other neurodegenerative disease in iNPH patients. Certain inflammatory proteins demonstrate an “inverse” expression pattern between disease entities, e.g., SERPINA3 and TIMP-1 upregulated in iNPH but reduced in Alzheimer’s disease, providing a potentially useful molecular distinction. This contrasting profile may support differential diagnosis in cases where clinical features overlap. Table 3 shows a comparison of levels of selected proteins in iNPH and its mimics relative to HC. The origin of iNPH may involve multiple distinct underlying mechanisms that converge into a similar clinical presentation; these differing etiologies may also account for variability in surgical response.

In an effort to identify potential indicators of response to shunting, some authors have proposed a panel of predictors [2,3]. However, the proteins listed are not consistent, indicating a need for further research to determine the most effective biomarkers.

The available data suggest that iNPH should be understood as a spectrum condition rather than a single disease entity. At one end of this spectrum, patients exhibit predominantly reversible axonal dysfunction associated with ventricular enlargement and mechanical stress. At the other end, patients may demonstrate a stronger neurodegenerative component, characterized by tau pathology and altered amyloid metabolism, often associated with poorer response to shunting.

#### 3.5.2. Clinical Implications

The main finding of this analysis is the identification of a potential “protein profile” based mainly on neurodegenerative (Aβ-42, T-tau, P-tau, NfL) and inflammatory proteins (SERPINA3, TIMP-1), which not only allows iNPH to be distinguished from other forms of neurodegenerative disorders, but may also serve as a key predictor of response to surgical treatment. In the context of prognosis after VPS the most important markers are T-tau and P-tau181. Low baseline T-tau concentration in CSF is associated with a more than threefold increase in the chance of gait improvement, while high T-tau levels (above 306 ng/L) correlate with a seventeen-fold increase in the risk of no cognitive improvement after surgery. In turn, high concentrations of P-tau suggest coexisting AD pathology, which usually predicts a poorer response to drainage. In contrast to classical neurodegenerative disorders, where elevated levels of neurofilament light (NfL) reflect irreversible axonal degeneration, increased NfL concentrations in iNPH are more likely to represent potentially reversible axonal injury driven by mechanical stretching and compression secondary to ventricular enlargement. This distinction is of particular translational importance, as it provides a mechanistic explanation for the seemingly paradoxical observation that higher baseline NfL levels may predict a more favorable response to VPS. Rather than indicating advanced neurodegeneration, elevated NfL in this context may reflect a state of dynamic, yet potentially reversible, axonal dysfunction that remains responsive to restoration of CSF dynamics. This supports its utility as both a potential biomarker of disease activity and a predictor of therapeutic reversibility.

iNPH is characterized by elevated levels of proinflammatory cytokines such as MCP-1 (CCL2) and CCL4, which distinguishes this disease from MCI and AD. The proteins SERPINA3 and TIMP-1 are of particular diagnostic value, as their concentrations are elevated in patients with iNPH, while they are reduced in the course of AD. In addition, impaired clearance of metabolites by the glymphatic system leads to the accumulation of Aβ, with a low Aβ-42/Aβ-40 ratio in CSF being a strong indicator of coexisting Alzheimer’s pathology, which complicates the diagnostic process. These findings should be placed in the broader context of knowledge about iNPH, which is evolving towards the perception of this entity as a complex neurodegenerative disease with a strong genetic basis. According to current knowledge, iNPH is a highly polygenic disease in which variants of genes such as Scm-like with four MBT domains protein 1 (SFMBT1), showing a loss of copy number in lining cells, and Cell Wall Biogenesis 43 C-Terminal Homolog (CWH43) [29] play an important role. These genetic predispositions affect the integrity of the BBB and the function of ependymal cilia, which is reflected in the observed proteomic changes, such as strong upregulation of the complement cascade (C1-C9 proteins) and acute phase proteins.

The variability of identified protein panels across studies highlights a fundamental limitation of proteomics, where high sensitivity results in detection of numerous statistically significant but not necessarily clinically actionable biomarkers. Therefore, CSF biomarkers should not be interpreted in isolation, but rather within a multidimensional profile reflecting the balance between reversible and irreversible brain injury, which may inform surgical decision making.

#### 3.5.3. Conceptual Framework: iNPH Vulnerability Model

On the basis of the current findings and the available evidence [30], we proposed iNPH proteomic vulnerability model, that should be interpreted as a hypothesis-generating conceptual framework rather than as a clinically validated tool. This model has been provided to synthesize the currently available, yet fragmented evidence on iNPH into a more coherent biological framework that may help explain the pathophysiological basis of the disease and variability in shunt responsiveness.

Within this framework, potentially reversible pathology is represented primarily by biomarker domains reflecting hydrocephalus-related mechanical and inflammatory dysfunction. These include axonal injury markers, particularly NfL, which in iNPH may reflect mechanical stretching and compression of periventricular axons; inflammatory and vascular markers, including MCP-1, CCL4, SERPINA3, TIMP-1, and related BBB-associated proteins; and selected extracellular matrix or cell adhesion proteins, such as DSG2, ITGB1, TGFBI, and related candidates, which may reflect disturbed tissue integrity and altered CSF–interstitial fluid dynamics. In this context, moderately elevated NfL in the absence of strong Alzheimer-type pathology may indicate active but potentially reversible axonal stress.

In contrast, relatively irreversible or less reversible pathology is proposed to be represented by biomarkers associated with established neurodegenerative burden. These include elevated P-tau181 and T-tau, a reduced Aβ42/Aβ40 ratio, and unfavorable synaptic or neuronal marker profiles suggestive of coexisting Alzheimer-type pathology or more advanced neuronal injury. Such a profile may reduce the likelihood of post-operative cognitive improvement, even when gait symptoms remain partially responsive to CSF diversion.

### 3.6. Limitations

Considerable inter-study heterogeneity was observed across the included studies, particularly with respect to cohort size, proteomic breadth, analytical platforms, and investigated biomarkers. Sample sizes ranged from 24 to 354 patients, while the number of analyzed proteins varied from targeted panels of several biomarkers to large-scale proteomic approaches assessing up to 2888 proteins. Smaller exploratory studies with broad proteomic screening may facilitate hypothesis generation and identification of novel molecular pathways; however, they are more susceptible to random variation and false-positive findings. In contrast, larger studies using targeted biomarker panels may provide more statistically robust and clinically interpretable results but have more limited discovery potential. These methodological differences reduce direct comparability between studies and limit the immediate clinical applicability of individual biomarkers. Furthermore, most included studies received weak global ratings in the EPHPP assessment, primarily due to their observational, non-randomized design and lack of reported blinding. Therefore, the conclusions of the present review should be interpreted with appropriate caution. Nevertheless, despite substantial methodological heterogeneity, several convergent biological patterns consistently emerged across independent studies, supporting the proposed integrative model of iNPH involving glymphatic dysfunction, neuroinflammation, blood–brain barrier impairment, and axonal stress. 

## 4. Materials and Methods

This review was conducted between 3 January and 10 February 2026 in accordance with the Preferred Reporting Items for Systematic Reviews and Meta-Analyses (PRISMA) guidelines. Ethical approval was not required due to the nature of the study design. The protocol was registered in the international PROSPERO database (Registration ID: CRD420261283971).

### 4.1. Literature Search and Eligibility Criteria

A systematic literature search was performed in PubMed, Web of Science, and Google Scholar to identify studies investigating proteomic biomarkers in idiopathic normal pressure hydrocephalus (iNPH). The following search strategy was applied: (“proteomics”) AND (“idiopathic normal pressure hydrocephalus” OR “iNPH”). Reference lists of relevant publications were additionally screened to identify potentially eligible studies. Studies were considered eligible if they fulfilled all of the following criteria: (1) original research articles published in English; (2) inclusion of adult human participants diagnosed with iNPH; and (3) application of proteomic analyses of CSF samples.

Studies were excluded if they: (1) involved animal models; (2) did not incorporate proteomic methodologies; or (3) constituted non-original publications, including review articles, book chapters, conference abstracts, and other forms of secondary literature. Clinical trials lacking primary proteomic data were also excluded. Review articles were not included in the systematic synthesis, but were used for the discussion of findings.

### 4.2. Study Selection and Data Extraction

**Figure 1:**
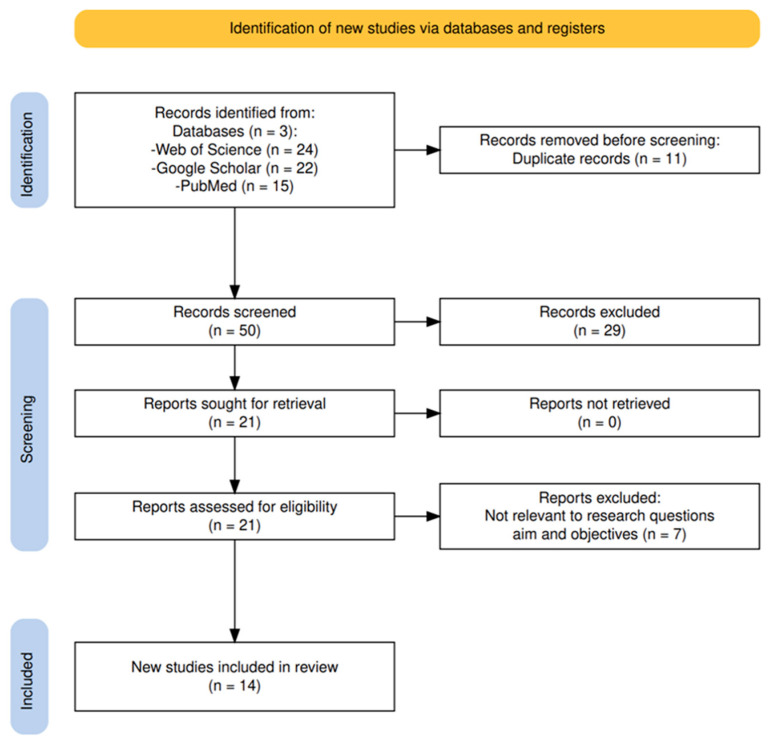
PRISMA flow chart.

Records retrieved from the literature search were screened after duplicate removal through sequential evaluation of titles, abstracts, and full-text articles according to predefined eligibility criteria. Screening, eligibility assessment, and data extraction were independently performed by three authors (A.K., A.L., and J.M.K.). Any disagreements regarding study selection or data extraction were resolved by consensus following consultation with a fourth author (M.D.). Reasons for exclusion at the full-text stage were documented and are presented in Supplementary Table S1. The study selection process is summarized in the PRISMA flow diagram (Figure 1) and in the Supplementary Material (PRISMA_2020_checklist). For each included study, the following information was extracted using a standardized form: first author and year of publication, study design and sample size, characteristics of the study population, biological material analyzed, proteomic platform and analytical methodology, identified proteins, and the main diagnostic and prognostic findings, including associations with shunt responsiveness.

### 4.3. Quality Assessment

The methodological quality of the included studies was assessed using the Effective Public Health Practice Project (EPHPP) Quality Assessment Tool for Quantitative Studies, as recommended by the National Collaborating Centre for Methods and Tools (NCCMT). The tool utilizes a systematic approach to evaluate the risk of bias across six domains: selection bias, study design, confounding, blinding, data collection methods, and withdrawals and dropouts. Each domain is rated as strong, moderate, or weak, and an overall methodological assessment (“Total assessment”) was derived for each study. The study’s findings were evaluated using the EPHPP criteria, a widely recognized framework for assessing the quality of health care providers. Assessments were conducted at the full-text level using reported methodological information. Given the exploratory and predominantly non-randomized nature of proteomic biomarker research, the results of the EPHPP assessment were interpreted within the context of observational biomarker discovery studies. Because substantial heterogeneity was anticipated with respect to study populations, proteomic platforms, and reported biomarkers, a quantitative meta-analysis was not planned. Therefore, findings were synthesized narratively, with particular emphasis on recurrent proteomic signatures, biological pathways implicated in iNPH pathophysiology, and the potential diagnostic and prognostic significance of identified biomarkers, including their relationship with VPS responsiveness. Moreover, based on these findings, a conceptual iNPH Proteomic Vulnerability Model has been proposed.

## 5. Conclusions

In conclusion, CSF proteomic studies provide biologically plausible insights into the pathophysiology of iNPH, revealing coherent but methodologically heterogeneous patterns involving inflammatory activation, BBB dysfunction, altered extracellular matrix dynamics, synaptic and neuronal marker changes, and axonal injury. Several proteins, including neurodegeneration-associated markers, inflammatory mediators, adhesion molecules, and selected prognostic candidates, have been associated with diagnostic differentiation or shunt responsiveness; however, these findings remain preliminary and require independent validation. Given the heterogeneity of clinical response to VPS, multidimensional protein signatures may offer greater explanatory and prognostic value than single biomarkers, particularly if they capture the balance between potentially reversible hydrocephalus-related dysfunction and less reversible neurodegenerative pathology. 

The conceptual iNPH Proteomic Vulnerability Model, while offering a unified approach for this multidimensional data, remains a preliminary proposal. Future research must, therefore, prioritize the rigorous statistical and clinical verification of this construct through extensive prospective cohort studies before it can be deployed in a clinical setting. Ultimately, research should focus on validating clinically applicable biomarker panels and incorporating proteomic data into models that reflect the balance between reversible and irreversible processes in iNPH.

## Figures and Tables

**Table 1:** Studies showing proteomics in iNPH.

Study [Reference]	Number of iNPH Patients	Mean Age [Years]	Main Findings	Shunting Response Assessment (Yes/No)
Braun et al., 2023 [7]	72	74 ± 6	MCP-1 and CCL4 levels were increased and PD-L1 level was reduced among patients with iNPH compared with CU and other neurodegenerative diseases.	No
De Geus et al., 2025 [6]	44	75 ± 7	BASP1, MDH1 and PKM showed AD-specific upregulation. NPTXR, NRXN3 and VGF were significantly downregulated, while F5 and LTBP2 were significantly upregulated in iNPH compared with AD and CU.	Yes
De Ryst et al., 2025 [11]	233	74 ± 6	iNPH patients presented AQP4 levels comparable to the control group, but lower preoperative AQP4 levels in shunt responders suggest a potential predictive role.	Yes
Ivarsson Orrelid et al., 2025 [12]	106	73 ± 8	There is a difference between l-CSF (more YWHAG protein) and v-CSF (more FABP3 protein) proteomic profile. Among the proteins studied, GOT1 shows the strongest correlation with the severity of pathological changes typical of AD in iNPH patients.	No
Kamalian et al., 2024 [1]	28	71 ± 5	A panel comprising 13 proteins has been identified as potential diagnostic biomarkers of iNPH.	Yes
Lukkarinen et al., 2021 [8]	39	75 ± 12	Neurodegenerative proteins: Aß-42, T-tau, P-tau 181, NfL, NRGN were examined before and after shunting. All biomarkers but Aß-42 increased notably by 140–810% in l-CSF after CSF diversion and then stabilized.	Yes
Lukkarinen et al., 2022 [9]	147	72 ± 7	Low CSF T-tau and absence of APOE ε4 predicted over 20% gait improvement postoperatively. Preoperative T-tau, P-tau 181 and NfL correlated negatively with MMSE scores both pre- and post-surgery.	Yes
Minta et al., 2021 [13]	31	74 ± 7	The increase in ECM proteins was observed. v-CSF TIMP-1 was inversely correlated with overall symptoms. High preoperative CSF NfL levels correlated with more favorable outcome after surgery.	Yes
Patel et al., 2024 [10]	354	78 ± 7	NPTX2 correlated with neurodegeneration, but not with cognitive function in iNPH. NPTX2 cannot serve as a SR predictor. Aβ42 had a prognostic value for iNPH. CSF P-tau-181 and Aβ40 concentrations were lower and the Aβ42/Aβ40 ratio was higher in the iNPH shunted group. Positive correlation between NPTX2 and P-tau-181 concentrations.	Yes
Rostgaard et al., 2023 [4]	62	75	Comm. and obs. differ in their protein profiles. No proteins were found to be a marker of SR in Comm. In Obs. a panel of 10 potential protein predictors was identified.	Yes
Rostgaard et al., 2023 [5]	46	75	Biomarkers of neurodegeneration are distributed in a specific pattern between the ventricles and the spinal canal: S100B, T-tau and P-tau levels are higher in the v-CSF; NfL, Aβ40, and Aβ42 are higher in l-CSF.	No
Torretta et al., 2021 [14]	24	86	iNPH patients’ protein profile is characterized by an increase in acute-phase proteins, immunoglobulins and complement component fragments. Proteins involved in synaptic signaling, axogenesis and proteins involved in lipid metabolism were statistically lower in iNPH than HC.	No
Weiner et al., 2023 [2]	68	74	FABP3, MIF, ANXA4, B3GAT2, ITGB1, YWHAG, OLFM2, TGFBI and DSG2 are promising prognostic biomarker candidates to predict SR in iNPH patients.	Yes
Ying et al., 2025 [3]	49	72 ± 6	Noticeably increased proteins were mainly associated with myeloid leukocyte migration and ECM organization, significantly decreased proteins with axon development and synapse organization. QPCT and RBP4 levels could potentially be SR predictors.	Yes

Abbreviations: MCP-1—monocyte chemoattractant protein-1, CCL4—C-C motif chemokine 4, PD-L1—programmed death ligand 1, BASP1—brain acid soluble protein 1, MDH1—malate dehydrogenase 1, PKM—pyruvate kinase, AD—Alzheimer’s disease, NPTXR—neuronal pentraxin receptor, NRXN3—neurexin 3, VGF—VGF nerve growth factor inducible, iNPH—idiopathic normal pressure hydrocephalus, CU—cognitively unimpaired, F5—coagulation factor 5, LTBP2—latent-transforming growth factor beta-binding protein 2, AQP4—aquaporin-4, YWHAG—14-3-3 protein gamma, CSF—cerebrospinal fluid, FABP3—heart-type fatty acid-binding protein, MSTN—myostatin, GOT1—Glutamic-Oxaloacetic Transaminase 1, CAMK2G—calcium/calmodulin-dependent protein kinase II gamma, Aß-42—Amyloid-β 42, T-tau—total tau, P-tau 181—phosphorylated tau (P-tau) at threonine 181, NfL—neurofilament light, NRGN—neurogranin, L-CSF—lumbar cerebrospinal fluid, MMSE—Mini-Mental State Examination, ECM—extracellular matrix, NPTX2—neuronal pentraxin-2, VIM—vimentin, PCDHAC2—protocadherin alpha-C2, GSS—glutathione synthetase, P4HB—Prolyl 4-Hydroxylase Subunit Beta, SDCBP—Syndecan Binding Protein, Obs—obstructive hydrocephalus, Comm—communicating hydrocephalus, CP—ceruloplasmin, CTSD—cathepsin D, HEXB—beta-hexosaminidase subunit beta, HPX—hemopexin, SERPINF1—pigment epithelium-derived factor, SERPINA3—serpin family A member 3, SIA—Sialate-O-acetylesterase, FN1—fibronectin 1, SELENOM—selenoprotein M, MIF—Macrophage Migration Inhibitory Factor, ANXA4—annexin A4, B3GAT2—Galactosylgalactosylxylosylprotein 3-beta-glucuronosyltransferase 2, ITGB1—Integrin subunit beta 1, YWHAG—14-3-3 protein gamma, OLFM2—olfactomedin 2, TGFBI—Transforming Growth Factor-Beta-Induced, DSG2—desmoglein-2, QPCT—glutaminyl-peptide cyclotransferase, RBP4—Retinol-Binding Protein 4, SR—shunt responsiveness.

**Table 2:** Methodological quality assessment of included studies using the EPHPP tool (the Effective Public Health Practice Project).

Study	Selection Bias	Design	Confounders	Blinding	Data Collection	Withdrawals and Dropouts	Total Assessment
**Braun et al., 2023** [7]	Moderate	Moderate	Strong	Weak	Strong	Moderate	**Moderate**
**De Geus et al., 2025** [6]	Moderate	Moderate	Strong	Weak	Strong	Moderate	**Moderate**
**De Ryst et al., 2025** [11]	Moderate	Moderate	Strong	Weak	Strong	Moderate	**Weak**
**Ivarsson Orrelid et al., 2025** [12]	Moderate	Moderate	Moderate	Weak	Strong	Moderate	**Weak**
**Kamalian et al., 2024** [1]	Moderate	Moderate	Strong	Weak	Strong	Moderate	**Moderate**
**Lukkarinen et al., 2021** [8]	Moderate	Moderate	Strong	Weak	Strong	Moderate	**Weak**
**Lukkarinen et al., 2022** [9]	Moderate	Moderate	Strong	Weak	Strong	Moderate	**Weak**
**Minta et al., 2021** [13]	Moderate	Moderate	Moderate	Weak	Strong	Moderate	**Weak**
**Patel et al., 2024** [10]	Moderate	Moderate	Strong	Weak	Strong	Moderate	**Weak**
**Rostgaard et al., 2023 (FBCNS)** [5]	Moderate	Moderate	Moderate	Weak	Strong	Moderate	**Weak**
**Rostgaard et al., 2023 (Acta Neurochir)** [4]	Moderate	Moderate	Moderate	Weak	Strong	Moderate	**Weak**
**Torretta et al., 2021** [14]	Moderate	Moderate	Moderate	Weak	Strong	Moderate	**Weak**
**Weiner et al., 2023** [2]	Moderate	Moderate	Strong	Weak	Strong	Moderate	**Weak**
**Ying et al., 2025** [3]	Moderate	Moderate	Strong	Weak	Strong	Moderate	**Weak**

**Table 3:** Comparison of levels of selected proteins in iNPH and its mimics relative to healthy controls.

Protein	iNPH	MCI	AD	Parkinsonism	HC	Studies Mentioning Protein (Reference)	Integrity of the Results
**APP**	↓	↑	↑	No data	Statistically higher than in iNPH, lower than in AD; age dependent	2, 3, 9, 10, 12, 18	Yes
**Aβ42/Aβ40** **ratio**	Average/↑	↓	↓ (threshold value: ratio < 0.0818)	No data	Average ratio = 0.11 (+/−0.014)	1, 6, 8, 10	Yes
**T-tau**	N/↓	N/↑	↑/↑↑	N/↑	N < 350 ng/L	2, 3, 5, 6, 7, 8, 9, 11, 12, 18	Yes
**P-tau**	N/↓	N/↑	↑	No data	N < 50 ng/L	1, 2, 3, 5, 6, 7, 8, 9, 10, 11, 12, 18	Yes
**Alpha-synuclein**	N	N	N	↑	N	1, 3, 7, 8, 10, 22	Yes
**NfL**	Average level/↑	↑	↑	No data	Average level = 2200 (+/−830) pg/mL	1, 2, 3, 5, 6, 7, 8, 9, 10, 11, 12, 18, 22	Yes
**Serpina3**	↑	No data	↓	No data	Significantly lower than in iNPH and higher than in AD	4, 12	Yes
**TIMP-1**	↑	No data	↓	No data	No established standard	12, 18	Yes
**MCP-1 (CCL2)**	↑↑	↑ Statistically lower than in iNPH	↑ Statistically lower than in iNPH	No data	Statistically lower than in iNPH	1, 3, 7, 9, 12	Yes
**CCL4**	↑↑	↑/↑↑	↑	↑	Statistically lower than in iNPH	1, 6, 7	Yes
**PD-L1**	↓	↓/↑	↑	No data	Statistically higher than in iNPH; age dependent	1, 6, 7	Yes
**L1CAM**	↓	Statistically higher than in iNPH	↓/↓↓	No data	Statistically higher than in iNPH	1, 3, 6, 12	Yes
**AQP4**	Comparable to HC	No data	↑	No data	Average level = 1351 (+/−279) AU	1, 2, 6, 7, 11, 12	Yes
**Vimentin**	↑	Statistically lower than in iNPH	Statistically lower than in iNPH	No data	Statistically lower than in iNPH	1, 3, 4	No
**LINGO1**	↓	No data	No data	↑	Statistically higher than in iNPH	3	Yes

Abbreviations: iNPH—idiopathic Normal Pressure Hydrocephalus, MCI—mild cognitive impairment, AD—Alzheimer’s disease, HC—healthy control, APP—Amyloid Precursor Protein, Aβ42—amyloid-β 42, Aβ40—amyloid-β 40, T-tau—total microtubule-associated protein tau, P-tau—phosphorylated microtubule-associated protein tau, NfL—neurofilament light polypeptide, MCP-1 (CCL2)—Monocyte Chemoattractant Protein-1, CCL4—C-C motif chemokine ligand 4, TIMP-1—TIMP metalloproteinase inhibitor 1, L1CAM—L1 cell adhesion molecule, LINGO1—leucine-rich repeat and immunoglobulin-like domain-containing nogo receptor-interacting protein 1. An upward arrow (↑) indicates that the analyzed protein concentration was higher in the particular group compared with healthy controls, whereas a downward arrow (↓) indicates that the reported protein concentration was lower compared with healthy control individuals.

## Data Availability

No new data were created or analyzed in this study.

## Supplementary Materials

The following supporting information can be downloaded at: https://www.mdpi.com/article/10.3390/molecules31132319/s1, PRISMA_2020_checklist; Table S1. Excluded articles and reasons for exclusion.

## Abbreviations

The following abbreviations are used in this manuscript:

Abbreviation	Definition
Aβ	Amyloid-β
Aβ38	Amyloid-β 38
Aβ40	Amyloid-β 40
Aβ42	Amyloid-β 42
AD	Alzheimer’s disease
ANXA4	Annexin A4
AQP4	Aquaporin-4
APP	Amyloid precursor protein
APOE	Apolipoprotein E
APOH	Apolipoprotein H
BASP1	Brain acid soluble protein 1
B3GAT2	Galactosylgalactosylxylosylprotein 3-beta-glucuronosyltransferase 2
BBB	Blood–brain barrier
BACE1	Beta-secretase 1
CAMK2G	Calcium/calmodulin-dependent protein kinase II gamma
CCL2	C-C motif chemokine ligand 2
CCL4	C-C motif chemokine 4
CCL11	Chemokine ligand 11
CDH2	N-cadherin
CDH5	Cadherin 5
CELSR2	Cadherin EGF LAG seven-pass G-type receptor 2
CHGA	Chromogranin A
CNS	Central nervous system
CP	Ceruloplasmin
CSF	Cerebrospinal fluid
CTSD	Cathepsin D
CU	Cognitively unimpaired
DSG2	Desmoglein-2
ECM	Extracellular matrix
EPHPP	Effective Public Health Practice Project
FABP3	Fatty acid binding protein 3
F5	Coagulation factor V
FN1	Fibronectin 1
GFAP	Glial fibrillary acidic protein
GOT1	Glutamic-Oxaloacetic Transaminase 1
GSS	Glutathione synthetase
HEXB	Beta-hexosaminidase subunit beta
HP	Haptoglobin
HPX	Hemopexin
iNPH	Idiopathic normal pressure hydrocephalus
ITGB1	Integrin subunit beta 1
L1CAM	L1 cell adhesion molecule
LCAT	Lecithin-cholesterol acyltransferase
L-CSF	Lumbar cerebrospinal fluid
LINGO1	Leucine-rich repeat and immunoglobulin-like domain-containing nogo receptor-interacting protein 1
LTBP2	Latent-transforming growth factor beta-binding protein 2
MAC	Membrane attack complex
MCP-1	Monocyte chemoattractant protein-1
MCI	Mild cognitive impairment
MDH1	Malate dehydrogenase 1
MIF	Macrophage migration inhibitory factor
MMSE	Mini-Mental State Examination
MMP	Matrix metalloproteinase
MSTN	Myostatin
NCCMT	National Collaborating Centre for Methods and Tools
NfL	Neurofilament light polypeptide
NPTX1	Neuronal pentraxin 1
NPTX2	Neuronal pentraxin 2
NPTXR	Neuronal pentraxin receptor
NRGN	Neurogranin
NRXN3	Neurexin 3
OLFM2	Olfactomedin 2
P4HB	Prolyl 4-hydroxylase subunit beta
PCDHAC2	Protocadherin alpha-C2
PD	Parkinson’s disease
PD-L1	Programmed death ligand 1
PKM	Pyruvate kinase
P-tau	Phosphorylated tau
P-tau181	Phosphorylated tau at threonine 181
QPCT	Glutaminyl-peptide cyclotransferase
RBP4	Retinol-binding protein 4
SCG2	Secretogranin 2
SCG3	Secretogranin 3
SDCBP	Syndecan binding protein
SELENOM	Selenoprotein M
SERPINA3	Serpin family A member 3
SERPINF1	Pigment epithelium-derived factor
SIA	Sialate-O-acetylesterase
SNAP25	Synaptosomal-associated protein 25
SR	Shunt responsiveness
SYT1	Synaptotagmin-1
TGFBI	Transforming growth factor-beta-induced
TIMP-1	Tissue inhibitor of metalloproteinases-1
T-tau	Total tau
VCAM1	Vascular cell adhesion protein 1
VGF	VGF nerve growth factor inducible
VIM	Vimentin
VPS	Ventriculoperitoneal shunting
