# Supplementary material for: Decoding the CSF Proteomic Signature of Idiopathic Normal Pressure Hydrocephalus: A Systematic Review
Source: Molecules. 2026 Jul 2;31(13):2319. doi: 10.3390/molecules31132319 (PMC13363446; doi:10.3390/molecules31132319)
Supplement: Supplementary file 1 [file molecules-31-02319-s001.zip › molecules-4330000-supplementary.pdf]

Table S1. Excluded articles and reasons for exclusion.

No.	Excluded article	Reason for exclusion
1	Guzelcicek, A.; Gönel, A.; Koyuncu, I.; Cigdem, G.; Kose, D.; Karadag, M.; Cadirci, D. Investigating the levels of brain-specific proteins in hydrocephalus patients. *Comb. Chem. High Throughput Screen.* **2021**, *24*, 409–414. https://doi.org/10.2174/1386207323666200720093245.	Non-adult cohort
2	Hale, A.T.; Zhou, B.; Rajan, A.; Duy, P.Q.; Goolam, M.; Alper, S.L.; Lehtinen, M.K.; Lancaster, M.A.; Fame, R.M.; Kahle, K.T. Molecular hallmarks of hydrocephalus. *Sci. Transl. Med.* **2025**, *17*, eadq1810. https://doi.org/10.1126/scitranslmed.adq1810.	Review
3	Li, J.; Zhang, X.; Guo, J.; Yu, C.; Yang, J. Molecular mechanisms and risk factors for the pathogenesis of hydrocephalus. *Front. Genet.* **2022**, *12*, 777926. https://doi.org/10.3389/fgene.2021.777926.	Review
4	Schirinzi, T.; Sancesario, G.M.; Di Lazzaro, G.; D'Elia, A.; Imbriani, P.; Scalise, S.; Pisani, A. Cerebrospinal fluid biomarkers profile of idiopathic normal pressure hydrocephalus. *J. Neural Transm.* **2018**, *125*, 673–679. https://doi.org/10.1007/s00702-018-1842-z.	No proteomic studies
5	van Gool, A.J.; Hendrickson, R.C. The proteomic toolbox for studying cerebrospinal fluid. *Expert Rev. Proteomics* **2012**, *9*, 165–179. https://doi.org/10.1586/epr.12.6.	Review
6	Yang, D.; Yang, H.; Luiselli, G.; Ogagan, C.; Dai, H.; Chiu, L.; Carroll, R.S.; Johnson, M.D. Increased plasmin-mediated proteolysis of L1CAM in a mouse model of idiopathic normal pressure hydrocephalus. *Proc. Natl. Acad. Sci. USA* **2021**, *118*, e2010528118. https://doi.org/10.1073/pnas.2010528118.	Animal model
7	Yuan, X.; Desiderio, D.M. Proteomics analysis of human cerebrospinal fluid. *J. Chromatogr. B Analyt. Technol. Biomed. Life Sci.* **2005**, *815*, 179–189. https://doi.org/10.1016/j.jchromb.2004.06.044.	No iNPH patients
